# Suppression of the Swallowing Reflex during Rhythmic Jaw Movements Induced by Repetitive Electrical Stimulation of the Dorsomedial Part of the Central Amygdaloid Nucleus in Rats

**DOI:** 10.3390/life10090190

**Published:** 2020-09-10

**Authors:** Yoshihide Satoh, Kojun Tsuji

**Affiliations:** Department of Physiology, The Nippon Dental University School of Life Dentistry at Niigata, 1-8 Hamaura-cho, Chuou-ku, Niigata 951-8580, Japan; baystars.v1.1998@gmail.com

**Keywords:** swallowing reflex, superior laryngeal nerve, rhythmic jaw movements, central amygdaloid nucleus

## Abstract

A previous study indicated that the swallowing reflex is inhibited during rhythmic jaw movements induced by electrical stimulation of the anterior cortical masticatory area. Rhythmic jaw movements were induced by electrical stimulation of the central amygdaloid nucleus (CeA). The swallowing central pattern generator is the nucleus of the solitary tract (NTS) and the lateral reticular formation in the medulla. Morphological studies have reported that the CeA projects to the NTS and the lateral reticular formation. It is therefore likely that the CeA is related to the control of the swallowing reflex. The purpose of this study was to determine if rhythmic jaw movements driven by CeA had inhibitory roles in the swallowing reflex induced by electrical stimulation of the superior laryngeal nerve (SLN). Rats were anesthetised with urethane. The SLN was solely stimulated for 10 s, and the swallowing reflex was recorded (SLN stimulation before SLN + CeA stimulation). Next, the SLN and the CeA were electrically stimulated at the same time for 10 s, and the swallowing reflex was recorded during rhythmic jaw movements (SLN + CeA stimulation). Finally, the SLN was solely stimulated (SLN stimulation following SLN + CeA stimulation). The number of swallows was reduced during rhythmic jaw movements. The onset latency of the first swallow was significantly longer in the SLN + CeA stimulation than in the SLN stimulation before SLN + CeA stimulation and SLN stimulation following SLN + CeA stimulation. These results support the idea that the coordination of swallowing reflex with rhythmic jaw movements could be regulated by the CeA.

## 1. Introduction

Rhythmic jaw movements were induced by repetitive electrical stimulation of the anterior and posterior cortical masticatory areas (CMA) in rats. The anterior area is the orofacial motor cortex, and the posterior area is the insular cortex [1,2,3]. Detailed analyses have been made of rhythmic jaw movements induced by stimulation of the CMA. The reciprocal connection between the anterior area and the posterior area of CMA is weak, and they work independently of each other [1,3]. Rhythmic jaw movements have been thought to be programmed by the masticatory central pattern generator (CPG) in the brainstem [4,5]. 

When a human masticates a solid food, part of the food is transported from the oral cavity to the oropharynx. Then, when a food bolus is accumulated in the oropharynx, swallowing reflex is induced. Mastication is continued until swallowing reflex is induced [6,7]. Swallowing movements are produced by the swallowing CPG in the medulla. The swallowing CPG is divided into two regions. The first region is the nucleus of the solitary tract (NTS) and the adjacent reticular formation. The second region is the lateral reticular formation above the nucleus ambiguous (LRF). The NTS works by initiating swallowing and programming the swallowing event. The LRF receives input from the NTS, and sends outputs to the motoneurons related to the swallowing [8,9,10]. 

A previous study found that the swallowing reflex is inhibited during rhythmic jaw movements induced by stimulation of the anterior CMA, whereas the swallowing reflex is not modulated during rhythmic jaw movements induced by stimulation of the posterior CMA in the rat [11]. This study suggests that the swallowing CPG is inhibited by the anterior CMA via the masticatory CPG.

The central amygdaloid nucleus (CeA) has been reported to have a role in fear [12,13], anxiety [14], increasing reward saliency [15,16], modulating food consumption [16,17], cardiovascular control and stress responses [18,19,20]. Rhythmic jaw movements are also induced by stimulation of the CeA in rats [21,22,23]. No detailed analysis of rhythmic jaw movements induced by stimulation of the CeA has been made, however. Electrophysiological and morphological studies have reported that the CeA projects directly to the NTS [24,25,26,27,28]. Furthermore, the CeA projects to the LRF [28,29]. It is therefore likely that the CeA is related to the control of the swallowing reflex. The purpose of the present study was to determine whether rhythmic jaw movements driven by CeA outflows had inhibitory roles on the swallowing reflex. 

## 2. Materials and Methods

These experiments were carried out using 7 male Sprague-Dawley rats weighing 327–380 g. All animal procedures followed the National Institute of Health Guide for the Care and Use of Laboratory Animals, and were approved by the Laboratory Animal Committee of The Nippon Dental University School of Life Dentistry at Niigata (approval number 186). 

The rats were initially anesthetized with urethane (1.3 g/kg, i.p.). Urethane (0.2 g/kg, i.v.) (Sigma-Aldrich Japan, Tokyo, Japan) was injected additionally via a cannula placed into the femoral vein when the withdrawal reflex was evoked by noxious stimulation of the hindlimb. Lidocaine (2% solution) (AstraZeneca, Osaka, Japan) was injected into the skin to minimize surgical pain before the incisions were made. The trachea was cannulated. Arterial blood pressure from the left femoral artery was monitored in order to confirm the condition of the rat until the end of the experiment. The arterial blood pressure was more than 70 mmHg and was stable. The rectal temperature was maintained at 37 °C by a heating pad and by the heat of the light (ATB-1100, Nihon Kohden, Tokyo, Japan). 

A midline incision was made along the ventral aspects from the pogonion to the caudal portion of the neck in supine position. The electromyogram (EMG) was recorded from the left mylohyoid (Myl) muscle by use of pairs of Teflon-coated silver wires (diameter 0.1 mm, exposed tip 2.0 mm) (Intermedical Co., Ltd., Nagoya, Japan). Jaw movements were recorded in the vertical and horizontal direction by a photodiode transducer that tracked the displacement of a light attached to the mandibular. The tip of bipolar silver wire electrodes (0.1 mm in diameter) for stimulation were bent and hooked bilaterally in the superior laryngeal nerve (SLN). The electrodes were fixed with silicon material, and were insulated from surrounding tissues. Repetitive electrical stimulation (0.2 ms duration, 30 Hz, 10 s) was applied to the SLN on the left or right side to evoke the swallowing reflex. The swallowing reflex was identified by visual observation of the laryngeal elevation, by the EMG burst in Myl muscles and by the larger jaw-closing movement than the jaw-closing movement during rhythmic jaw movements. The threshold of the SLN was decided to be the minimum stimulus intensity that could induce swallowing reflex at least once during 10 s. The SLN was stimulated at 1.2 times the threshold.

After fixation of the SLN electrode, the rat was inverted from the supine to prone position. The head of the rat was placed on a stereotaxic apparatus (SN-3S, Narishige Scientific Instrument Lab., Tokyo, Japan), using ear bars and an incisal bar. Part of the parietal bone was removed, using a dental drill to expose the cerebral cortex. A bipolar concentric electrode (outer diameter 200 μm) (TK213-091, Unique Medical Co., Ltd., Tokyo, Japan) was stereotaxically inserted into the left CeA. Rhythmic jaw movements were evoked by repetitive electrical stimulation of the CeA (0.2 ms duration, 30 Hz, 200–300 μA 10 s).

After confirming that rhythmic jaw movements were evoked, the SLN alone was stimulated for 10 s (SLN stimulation before SLN + CeA stimulation). Then, 1 min later, the SLN and the CeA were stimulated at the same time for 10 s to investigate whether the swallowing reflex is inhibited during rhythmic jaw movements (SLN + CeA stimulation). Then, 1 min later, the SLN alone was stimulated same as SLN stimulation before SLN + CeA stimulation (SLN stimulation following SLN + CeA stimulation) to confirm whether the number of swallows returned to the level of SLN stimulation before SLN + CeA stimulation. If the SLN threshold was continuously increased, the number of swallows during SLN + CeA stimulation became smaller than that during SLN stimulation before SLN + CeA stimulation. This means that the suppression of the swallowing reflex is not caused by rhythmic jaw movements and is caused by increase in the SLN threshold. Two trials were performed from SLN stimulation before SLN + CeA stimulation to SLN stimulation following SLN + CeA stimulation for each rat. The average of two trials was taken as the value for that rat.

The electromyographic responses to stimulation of the SLN and the CeA were amplified (filter bandwidth 10 Hz–1 kHz) and stored on a computer disk. Data were analyzed by the Spike2 analysis package version 7 (Cambridge Electronic Design, Cambridge, UK) at a sampling rate of 2 kHz. In each recording session we measured the number of swallows, the onset latency of the first swallow (defined as the time from the onset of SLN stimulus to the peak of the Myl EMG burst), the cycle time of jaw movements and frequency of rhythmic jaw movements. The cycle time of jaw movements was taken to begin at the moment of maximum jaw-closing, and to end at the next maximum jaw-closing. Control data were calculated as the average of two values for the number of swallows, and the onset latency of the first swallow. The Wilcoxon signed-rank test with Bonferroni correction, followed by Friedman’s test for a post hoc test, were used to test the effects of electrical stimulation on the CeA. Statistical differences were defined at the *p* < 0.05 level in all statistical tests.

After recording the swallowing reflex and the jaw movements, the animals were then given a lethal dose of anesthetic. Electrolytic lesions were made by passing a negative direct current (20 μA for 90 s) through the CeA-stimulating electrode. The brain was fixed in a 10% buffered formalin solution (pH 7.4). Serial coronal sections (60 μm thick) of the brain and Nissl staining were made. The sites of stimulation were checked histologically according to a rat brain map [30].

## 3. Results

### 3.1. Electrical Stimulation Sites in the CeA

**Figure 1:**
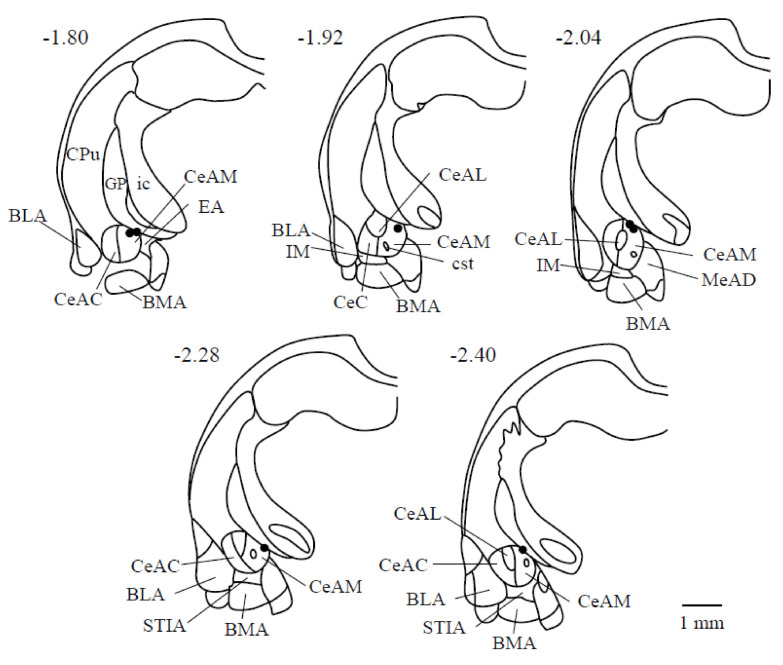
Electrical stimulation sites are shown of the limbic system in seven rats. The number of each section represents the level of its coronal section from the bregma in mm; (-) signs denote caudal to the bregma. The right direction of each drawing is the right side of the animal. Abbreviations: BLA: basolateral amygdaloid nucleus, anterior part; BMA: basomedial amygdaloid nucleus, anterior part; CeAC: central amygdaloid nucleus, capsular part; CeAM: central amygdaloid nucleus, medial division; CeAL: central amygdaloid nucleus, lateral division; CPu: caudate putamen (striatum); cst: commissural stria terminalis; EA: sublenticular extended amygdala; GP: globus pallidus; ic: internal capsule; IM: intercalated amygdaloid nucleus, main part; LaVM: lateral amygdaloid nucleus, ventromedial part; STIA: bed nucleus of the stria terminalis, intra-amygdaloid division.

Figure 1 shows the electrical stimulation sites in the CeA. The stimulation sites that induced rhythmic jaw movements are located in the dorsomedial part of the CeA (CeADM); stimulation of the ventrolateral part of the CeA or the other areas in the amygdala did not induce rhythmic jaw movements. 

**Figure 2:**
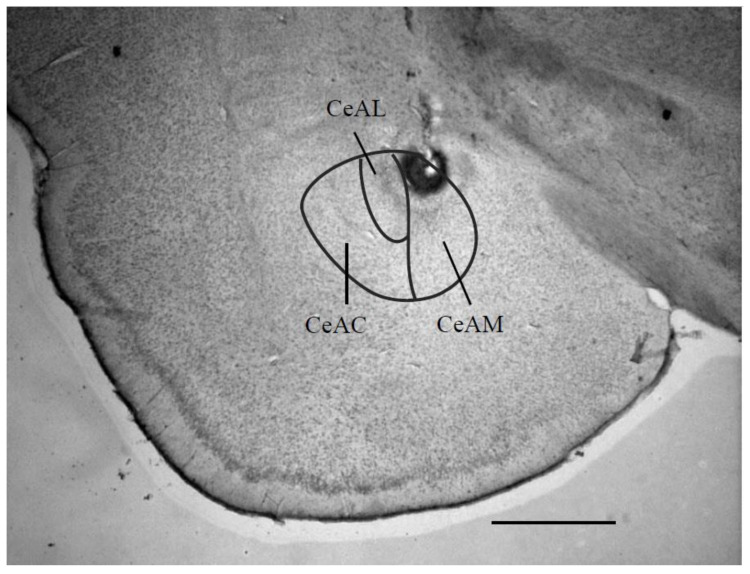
Photomicrograph of a coronal section through the limbic system, showing the site of the central amygdaloid nucleus stimulation. This section is -2.40 mm caudal to the bregma. Scale bar = 1 mm. Abbreviations: CeAC: central amygdaloid nucleus, capsular part; CeAM: central amygdaloid nucleus, medial division; CeAL: central amygdaloid nucleus.

Figure 2 indicates a photomicrograph of an electrical stimulation site in the left CeADM.

### 3.2. Rhythmic Jaw Movements 

**Figure 3:**
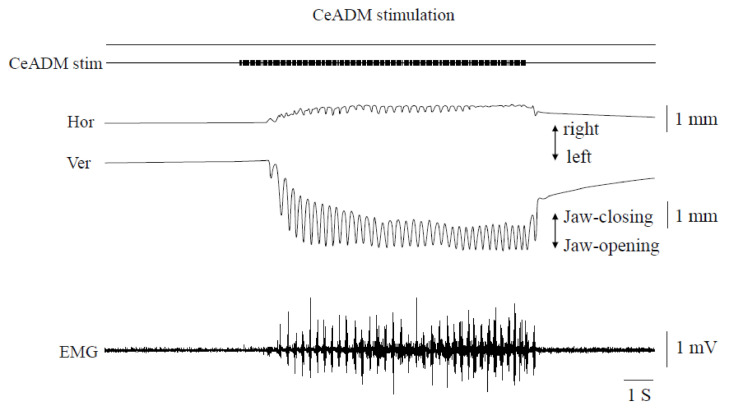
Jaw movement trajectories and muscle activities during rhythmic jaw movements induced by stimulating the left dorsomedial part of the central amygdaloid nucleus. Hor: horizontal jaw movements. Ver: vertical jaw movements. EMG: electromyographic activities of the mylohyoid muscle on the left side.

Rhythmic jaw movements were induced by stimulation of the CeADM (Figure 3). The threshold of the CeADM for inducing rhythmic jaw movements was 251.4 ± 31.3 μA (mean ± SD, *n* = 7). 

The movements always began with an opening of the jaw, followed by rhythmic movements consisting of simple opening-closing movements. The uppermost position during rhythmic jaw movements was below the rest position of the mandible. The horizontal jaw movements were very small and always began with a rightward movement. The cycle time of jaw movements was 219.7 ± 31.4 ms (mean ± SEM, *n* = 7). The frequency of rhythmic jaw movements was 4.9 ± 0.6 Hz (mean ± SEM, *n* = 7). Activity in the Myl muscle EMG manifested as bursts during the jaw-opening phase. The pattern of rhythmic jaw movements induced was similar in different rats.

### 3.3. Swallowing Reflex 

The threshold intensity to evoke swallowing reflex by stimulating the SLN was 234.3 ± 95.9 μA (mean ± SD, *n* = 7). Large jaw-closing movements occurred consistently with the swallowing reflex. The jaw was slightly open (i.e., mandibular rest position) in the prone position when the SLN was not stimulated. Accordingly, the upper and lower teeth did not contact even if the large jaw-closing movements occurred. The EMG burst in Myl muscles did not manifest with the jaw-opening phase during SLN + CeADM stimulation.

**Figure 4:**
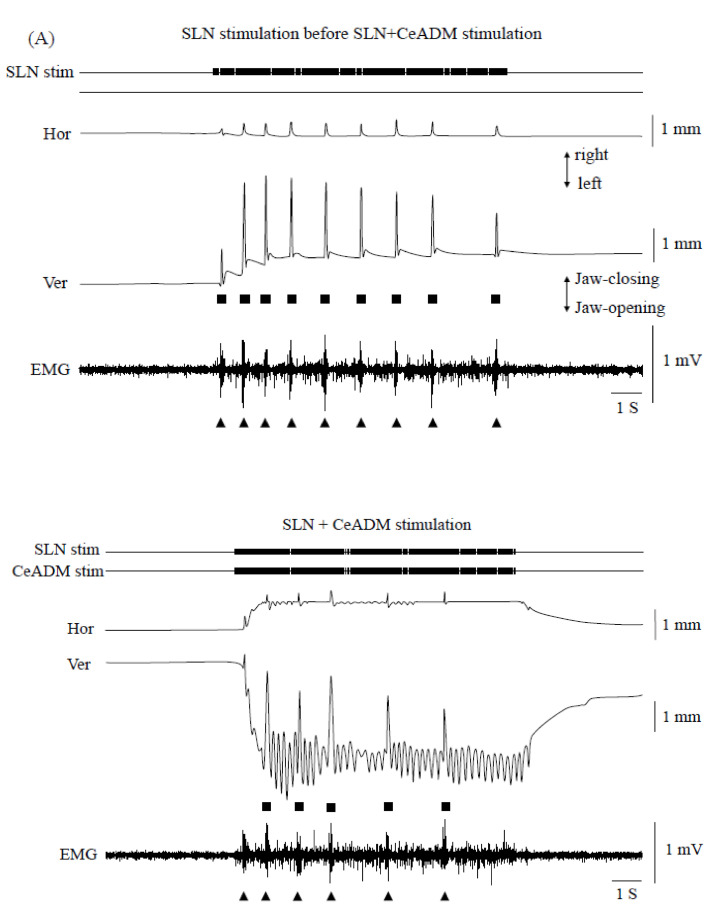

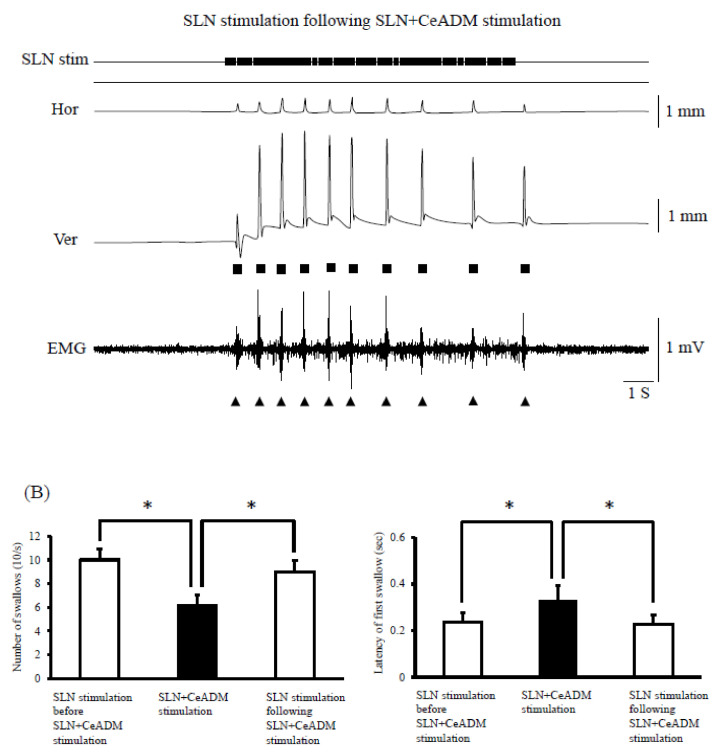
(**A**) Examples of jaw movements and electromyogram (EMG) activities during the superior laryngeal nerve (SLN) stimulation or the SLN + the dorsomedial part of the central amygdaloid nucleus (CeADM) stimulation. Each filled triangle indicates the occurrence of swallowing. Each filled square indicates the larger jaw-closing movement than the jaw-closing movement during rhythmic jaw movements. (**B**) Changes of the number of swallows (left) and of the onset latency of the first swallow (right) by electrical stimulation of the CeADM (*n* = 7). Vertical bars are ± SEM. Asterisks show significant difference (*p* < 0.05).

The swallowing reflexes were suppressed (*n* = 7) during rhythmic jaw movements (Figure 4). The number of swallows was significantly less during rhythmic jaw movements than with no rhythmic jaw movements (*p* = 0.003) (Figure 4B, left). The number of swallows was 10.0 ± 1.0 (mean ± SEM) in SLN stimulation before SLN + CeADM stimulation, 6.3 ± 0.8 during SLN + CeADM stimulation, and 9.0 ± 0.9 in SLN stimulation following SLN + CeADM stimulation (*n* = 7). The onset latency of the first swallow was significantly prolonged (*p* = 0.010) (Figure 4B, right). The onset latency of the first swallow was 0.23 ± 0.04 s (mean ± SEM) in SLN stimulation before SLN + CeADM stimulation, 0.33 ± 0.07 s during SLN + CeADM stimulation, and 0.23 ± 0.04 s in SLN stimulation following SLN + CeADM stimulation (*n* = 7).

The cycle time of jaw movements was 231.6 ± 32.7 ms (mean ± SEM, *n* = 7) during SLN + CeADM stimulation. The cycle time of jaw-movements was prolonged only when a large closing movement occurred simultaneously with the swallowing reflex. The cycle time of jaw-movements during SLN + CeADM stimulation was almost same as SLN stimulation before SLN + CeADM stimulation while the swallowing reflex was not induced. As a result, the cycle time of jaw-movements during SLN + CeADM stimulation was significantly longer than SLN stimulation before SLN + CeADM stimulation (*p* = 0.034). The frequency of rhythmic jaw movements was 4.3 ± 0.5 Hz (mean ± SEM, *n* = 7), and was not significantly changed during SLN + CeADM stimulation (*p* = 0.055).

The swallowing reflex was not significantly changed during the weaker stimulus intensity than the threshold for inducing the rhythmic jaw movements by repetitive electrical stimulation of the CeADM. The number of swallows was 10.1 ± 0.6 (mean ± SEM) in SLN stimulation before SLN + CeADM stimulation, 8.1 ± 0.5 during no rhythmic jaw movements, and 8.3 ± 0.9 in SLN stimulation following SLN + CeADM stimulation (*n* = 7, *p* = 0.261). The onset latency of the first swallow was 0.22 ± 0.03 s in SLN stimulation before SLN + CeADM stimulation (mean ± SEM), 0.27 ± 0.05 s during no rhythmic jaw movements, and 0.24 ± 0.04 s in SLN stimulation following SLN + CeADM stimulation (*n* = 7, *p* = 0.650).

## 4. Discussion

Of the seven data in this study, the threshold to evoke the swallowing reflex was higher in five of the data than that in previous studies [11,31,32]. When the head of animals was fixed using a stereotaxic apparatus, the rat was inverted from the supine to prone position. As a result, the threshold was increased by the flexion of the head in some cases. The SLN alone was stimulated following SLN + CeADM stimulation to confirm that the suppression of the swallowing reflex is caused by rhythmic jaw movements, and is not caused by the increase in the SLN threshold. If the number of swallows in SLN stimulation following SLN + CeADM stimulation was less than during SLN + CeADM stimulation, no data were collected because there is a possibility that the reduction in the number of swallows during rhythmic jaw movements is due to continuously increasing of the SLN threshold.

Properties of rhythmic jaw movements induced by the CeADM and the CMA are different. The frequency of rhythmic jaw movements was 3–5 Hz induced by the CeADM. However, the frequency of rhythmic jaw movements was 5–7 Hz and 3–4 Hz induced by the anterior and posterior CMA, respectively [1,2]. The location of the masticatory CPG of CMA and CeADM is therefore different. The previous study indicated that the number of swallows was less, but the onset latency of the first swallow was not changed during rhythmic jaw movements induced by stimulation of the anterior CMA in the rat. Tsujimura et al. suggested that swallowing inhibition may be mediated by inputs from the anterior CMA via the masticatory CPG into the swallowing CPG [11]. The location of the masticatory CPG of the CMA has been thought to be at the gigantocelluar reticular nucleus [4] or the dorsal part of the principal sensory trigeminal nucleus in the brainstem [5]. The swallowing reflex was not changed when the stimulus intensity of the CeADM was weak and the rhythmic jaw movements were not induced. Therefore, it is suggested that suppression of the swallowing reflex during rhythmic jaw movement is caused by inhibition of the masticatory CPG of CeADM to the swallowing CPG. 

This study demonstrated that the number of swallows was significantly less during rhythmic jaw movements induced by stimulation of the CeADM. The CeADM projects to the LRF [28,29]. It has been reported that the number of swallows induced by the SLN stimulation was reduced after microinjection of a GABA_A_ agonist into the LRF [33]. The CeADM contains GABAergic inhibitory interneurons [26,27,34]. GABAergic inputs from the CeADM and the masticatory CPG of CeADM may inhibit activation in the ventral region of swallowing CPG.

The onset latency of the first swallow was significantly prolonged during rhythmic jaw movements. A morphological study showed direct GABAergic projection from the CeADM to the NTS [26,27]. It is likely, therefore, that the direct input from the CeADM and the masticatory CPG of the CeADM to the NTS is involved in the extension of the onset latency of the first swallow by stimulation of the CeADM.

The CeA sends projections to the mesencephalic trigeminal nucleus, the supratrigeminal nucleus, and the spinal trigeminal nucleus caudalis [35,36,37]. The mesencephalic trigeminal nucleus and the supratrigeminal nucleus project to the LRF [38,39,40]. The spinal trigeminal nucleus caudalis projects to the NTS [41,42]. These areas may also therefore be involved in the suppression of the swallowing reflex during rhythmic jaw movements induced by stimulation of the CeADM.

Although rhythmic jaw movements were induced during SLN + CeADM stimulation, the EMG burst in Myl muscles was not manifested with the jaw-opening phase. The large jaw-closing movements that occurred were consistent with the swallowing reflex. It is assumed that the Myl muscle activity was affected by the induction of three types of movements during SLN + CeADM stimulation. The jaw-opening motoneurons may be inhibited by the large jaw-closing movement during the jaw-opening phase of rhythmic jaw movements or when the swallowing reflex is induced. On the other hand, the jaw-closing motoneurons may be inhibited during the jaw-opening phase and be excited during the jaw-closing phase. The NTS, which connects with the afferent terminals of the SLN, contains the premotor neurons projecting to the jaw-closing and jaw-opening motoneurons in the trigeminal motor nucleus [43]. It is possible that some of the premotoneurons projecting to the jaw-opening motoneurons in the NTS are inhibitory interneurons, since the NTS contains GABAergic neurons [44,45] and the jaw-opening reflex is suppressed by the swallowing reflex induced by the stimulation of the SLN [46]. The CeA projects to the mesencephalic trigeminal nucleus and the supratrigeminal nucleus as mentioned above [35,36,37]. The mesencephalic trigeminal nucleus has monosynaptic excitatory projections to the jaw-closing motoneurons [47,48]. The supratrigeminal nucleus contains the inhibitory premotoneurons projecting to the jaw-closing motoneurons [49,50]. In addition, the cycle time of jaw-movements during SLN + CeADM stimulation was significantly longer than SLN stimulation before SLN + CeADM stimulation in the present study. Therefore, there is the possibility that the swallowing CPG also influences the masticatory CPG of the CeA and/or the trigeminal motor nucleus. Further research is needed to clear this question. For example, recordings of the CeADM induced-jaw movements and of neuronal activities from the trigeminal motor nucleus during stimulation of the swallowing CPG are needed.

The jaw-closing muscles are stretched in the jaw-opening phase of the rhythmic jaw movements. As muscle spindles are richly found in the jaw-closing muscles [51], muscle spindles are excited during the jaw-opening phase. The mesencephalic trigeminal nucleus contains the cell bodies of primary and secondary afferents from muscle spindles of the jaw-closing muscles [47,52], and projects to the supratrigeminal nucleus [53]. Therefore, there is the possibility that the muscle spindles of the jaw-closing muscles also have a role in the suppression of the swallowing reflex.

In conclusion, the present study suggests that the CeADM and the masticatory CPG of the CeA affects the swallowing CPG, and also that the CeA may be involved in the emotional aspects of deglutition.
